# Control of Early Flame Kernel Growth by Multi-Wavelength Laser Pulses for Enhanced Ignition

**DOI:** 10.1038/s41598-017-10457-0

**Published:** 2017-08-31

**Authors:** Ciprian Dumitrache, Rachel VanOsdol, Christopher M. Limbach, Azer P. Yalin

**Affiliations:** 1 Colorado State University, Department of Mechanical Engineering, Fort Collins, 80523 USA; 2 Colorado State University, Department of Chemistry, Fort Collins, 80523 USA

## Abstract

The present contribution examines the impact of plasma dynamics and plasma-driven fluid dynamics on the flame growth of laser ignited mixtures and shows that a new dual-pulse scheme can be used to control the kernel formation process in ways that extend the lean ignition limit. We perform a comparative study between (conventional) single-pulse laser ignition (λ = 1064 nm) and a novel dual-pulse method based on combining an ultraviolet (UV) pre-ionization pulse (λ = 266 nm) with an overlapped near-infrared (NIR) energy addition pulse (λ = 1064 nm). We employ OH* chemiluminescence to visualize the evolution of the early flame kernel. For single-pulse laser ignition at lean conditions, the flame kernel separates through third lobe detachment, corresponding to high strain rates that extinguish the flame. In this work, we investigate the capabilities of the dual-pulse to control the plasma-driven fluid dynamics by adjusting the axial offset of the two focal points. In particular, we find there exists a beam waist offset whereby the resulting vorticity suppresses formation of the third lobe, consequently reducing flame stretch. With this approach, we demonstrate that the dual-pulse method enables reduced flame speeds (at early times), an extended lean limit, increased combustion efficiency, and decreased laser energy requirements.

## Introduction

Laser ignition is of interest as an alternative technology for a host of combustion applications including reciprocating engines, aero-turbines, and rocket engines^1–7^. Its flexible and non-intrusive nature may provide advantages over conventional igniters such as capacitive discharge spark ignition systems. The technique eliminates the presence of electrodes within the combustion chamber which tend to act as heat sinks leading to kernel extinction at lean conditions. Additionally, laser ignition can be achieved with very precise timing and with freedom in choosing the ignition location. By selecting appropriate focusing optics, the combustion initiation site can be moved away from combustor walls to regions where the mixture is more readily ignited, for example where droplet sizes, velocity and stoichiometry are favorable in the case of aero-turbine combustors^8–10^.

In addition to improved control over position and timing, the laser-induced plasma has fundamentally different conditions, such as temperature, density, size, and lifetime, as compared to other spark plug igniter plasmas. These micro-scale differences in the plasma kernel influence flame chemistry and flame dynamics, which in turn can expand the ignition window in practical combustion devices. In particular, a substantial body of research has investigated laser ignition of industrial reciprocating natural gas engines including showing extension of the lean limit and reduction in NO_x_ emissions^11–13^. Nonetheless, there are also certain challenges associated with laser ignition. The process tends to be energetically inefficient, for example, Phuoc *et al*. showed that only ~10% of the laser energy absorbed into the spark is available for ignition while most of the energy is lost through blast wave propagation and other dissipative processes^14^. From a practical point of view, commercial adoption has not yet occurred in part due to the need for fully reliable systems, potentially with fiber optic delivery^15–19^, which should be based on relatively inexpensive laser sources. There has been substantial recent progress towards appropriate sources, for example VCSEL pumped Nd:YAG lasers^20^ and ceramic microchip lasers^21, 22^. Improvements in plasma energy coupling, as we show with the dual-pulse approach, can benefit and expand the applicability of different laser sources to practical ignition systems.

There have been several studies of the fluid mechanic aspects of flame kernel formation under laser breakdown ignition. Morsy *et al*.^23^ showed numerically that a toroidal shaped flame kernel is formed in the wake of the shock wave prompted by the laser spark. Typically, the flame ignited using this method develops a front lobe that appears on the upstream (incident) laser side and propagates toward the laser source. The front lobe is often referred to as the third lobe, since the toroidal kernel resembles a two-lobe structure in two-dimensional cross-sectional images. The three lobe structure has been observed in air as well as both flammable and non-flammable mixtures^24–26^. Morsy *et al*. noted that it is possible for the third lobe to separate from the main flame kernel but do not discuss this in connection with flame quenching as we investigate here. The formation mechanism of the third lobe is first discussed by Bradley *et al*.^26^ who suggest that the third lobe forms due to an asymmetric inward flow induced by the passing rarefaction waves. The interaction between the rarefaction and the expanding hot gas behaves as a Taylor instability within the kernel, generating a pair of counter-rotating vortices–one at the upstream (laser incident) side and one at the downstream side of the kernel. Owing to the non-uniform energy addition around the beam waist (with more energy absorbed towards the laser^27^), the downstream vortex is stronger and forms the third lobe through the entrainment of the surrounding cold gas in an axial jet which impinges upon and expels the hot gases in the plasma core. Ghosh and Mahesh also discuss the dynamics of vorticity in laser-induced sparks as observed in their numerical simulations^28^. They suggest that at short time scales (prior to plasma recombination) vorticity is generated through a baroclinic torque induced in the flow by misaligned pressure and density gradients, while at longer time scales additional vorticity is created by roll-up of the plasma core (similar to the model of Bradley *et al*.). Similar mechanisms to those discussed here (in the context of laser ignition) are also responsible for kernel dynamics induced by conventional spark plugs and in nanosecond discharges between electrode pairs. However, in these cases the energy deposition is more symmetric so that two matched vortex rings persist and no third lobe forms^29–32^. Finally, Endo *et al*. have reported a comparative study between laser breakdown ignition and discharge spark plugs indicating that the plasma-driven fluid dynamics play an important role in flame kernel augmentation^24^. They suggest that flame vorticity entrains the surrounding combustible mixture which leads to an increase of the effective kernel energy in the early stages of flame development.

The research presented herein examines a novel laser ignition technique based on a dual-pulse pre-ionization scheme with nanosecond pulses. We use an ultraviolet (UV) pulse, at λ = 266 nm, to generate gas pre-ionization (not full breakdown) at the laser focal spot. The UV pulse is followed several nanoseconds later (Δτ = 15 ns) by an overlapped near-infrared (NIR) pulse (λ = 1064 nm) that adds energy into the ionized gas thereby increasing its temperature by several thousand degrees Kelvin (~2000–3000 K). The gas pre-ionization is obtained primarily through the multi-photon ionization (MPI) process, which is favored in the UV wavelength range, whereas the energy addition is primarily by inverse bremsstrahlung (IB) absorption, which is stronger in the NIR^27, 33–35^. The main difference between this new technique and conventional single-pulse laser ignition is that in the dual-pulse case the first pulse does not achieve full breakdown, rather it produces pre-ionization electrons that seed the electron avalanche process when illuminated by the IR pulse. This decoupling of plasma formation steps allows access to a larger space of plasma parameters, effectively providing a means of tailoring the plasma temperature and density. For example, while typical single-pulse laser sparks in air are fully ionized with temperatures in excess of 10,000K^36, 37^, the dual-pulse technique allows generation of plasmas with varying ionization fraction (between ~0.0001 and 0.01) within a wide range of temperatures (between ~400 and 10,000 K)^37^. The use of pre-ionization for laser ignition of methane-air mixtures was also demonstrated by Michael *et al*.^38^. In their work, seed electrons were generated by a femtosecond laser pulse with a subcritical microwave pulse providing energy addition. Dual-pulse approaches have also been examined in laser induced breakdown spectroscopy (LIBS), generally to enhance signal levels, but in configurations where the first pulse produces fully ionized plasma^39^.

When the decoupled dual-pulse scheme is applied towards ignition in fuel-air mixtures, the ensuing flame kernels exhibit differences in their fluid mechanics, shape, and growth which ultimately result in combustion benefits relative to conventional laser ignition. As shown herein, experiments comparing propane-air ignition with dual-pulses and single-pulses demonstrate that proper adjustment of the dual-pulse technique results in an increase in combustion efficiency for lean mixtures, extension of lean limit, and reduction in the total (UV plus NIR) laser pulse energy required for ignition. The nature of the flame kernel growth is elucidated by OH* chemiluminescence (CL) imaging which shows that, for both single- and dual-pulse methods, the flame dynamics are dictated by the flow induced during the plasma recombination process. For single-pulse ignition, the third lobe always grows towards the incident beam direction, and its detachment correlates with quenching of the flame kernel. For the dual-pulse method, we find that varying the axial offset of the UV and NIR focal spot positions (by ~1–3 mm) yields qualitatively different flame kernel structures in which the third lobe can be formed at either the upstream (laser incident) side or downstream side of the kernel, or not at all. The CL images indicate that suppression of the third lobe leads to more robust flame kernel growth, and it is for these conditions that the dual-pulse method shows extension of the lean limit.

## Results and Discussions

### Single-Pulse Laser Ignition

**Figure 1:**
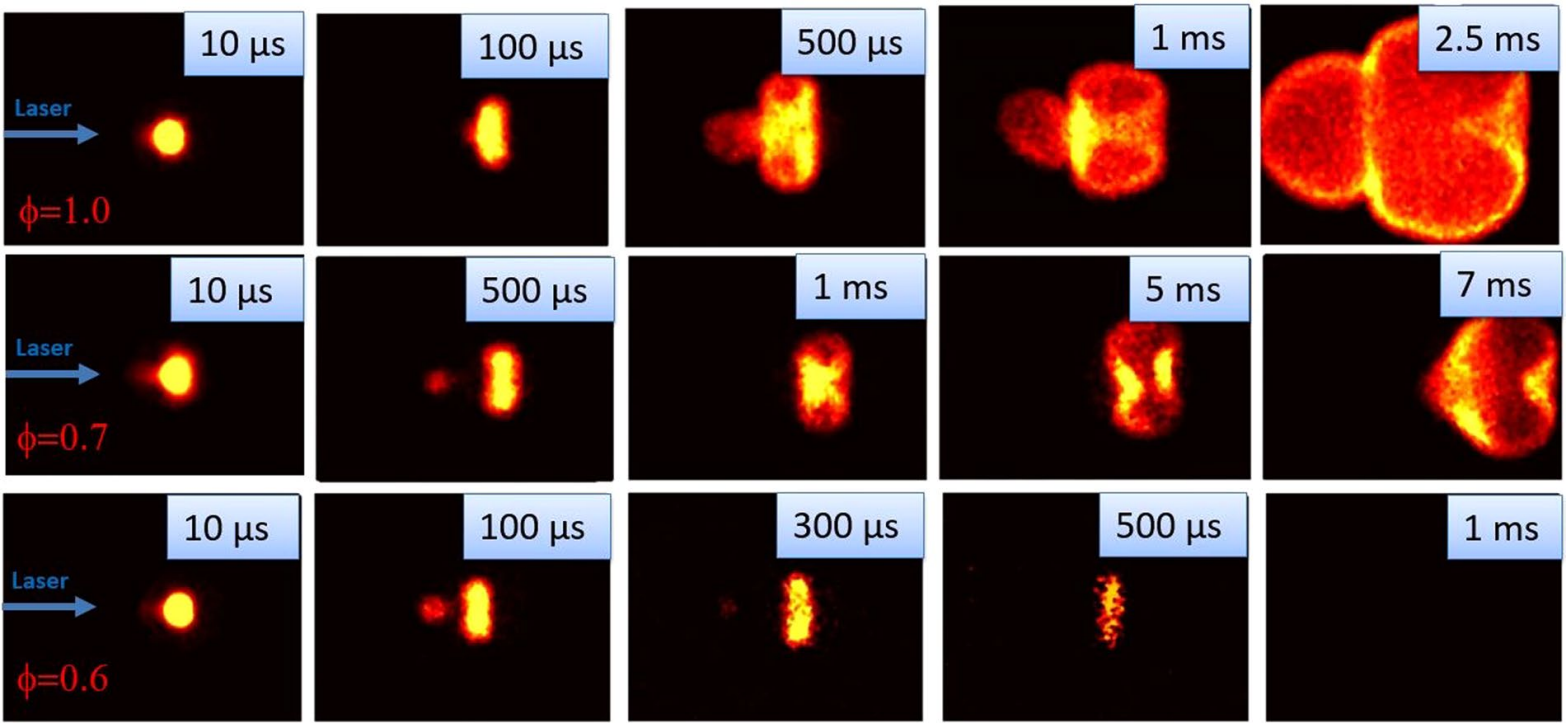
Flame kernel development during single-pulse ignition of propane air mixtures at ϕ = 1.0 (top), ϕ = 0.7 (middle), and ϕ = 0.6 (bottom). The third lobe separates from the main kernel and is a precursor to flame quenching for the lean mixtures. Each image frame has field of view: 22 mm × 16 mm.

The first set of experiments investigated ignition of propane-air mixture using single NIR laser pulses, i.e. conventional laser ignition approach. The NIR laser energy was set to *E* = 75 mJ to ensure reliable laser plasma formation, i.e. “sparking” with 100% probability. In this way, any non-combusting cases are due to problems with flame kernel growth, not simply lack of an initiation spark. OH* chemiluminescence images collected at various equivalence ratios are presented in Fig. 1. The top row of images show flame kernel development and propagation at ϕ = 1.0. Under these conditions, one can clearly observe the toroidal flame structure that was previously reported by other researchers as well as the presence of the third lobe on the upstream side^26^. The flame dynamics in this case are largely dictated by the flow vorticity induced during plasma recombination and cooling^23, 26^. At leaner conditions, for example in the middle row of images for which ϕ = 0.7, the third lobe detaches causing the flame kernel to break into two separate flamelets. The splitting (and in some cases extinction) of the kernel at lean conditions is attributed to insufficient energy deposition in the third lobe given the flame stretching induced by the vorticity effects of the laser plasma; it can be considered as a consequence of the increase in Karlovitz number with the decrease in equivalence ratio^26^. At even leaner conditions, i.e. the bottom row of images for which ϕ = 0.6, the flame kernel never develops into a self-propagating flame.

The image sequences suggest a relationship between the moment of lobe separation and ensuing flame quenching. In the lean mixtures cases, one can observe that the separation of the third lobe negatively impacts the rate of growth of the remaining flame kernel. For example, for ϕ = 0.7, the kernel at 5 ms is observed to be similar in size with that at ~1 ms (or earlier) for ϕ = 1.0. The third lobe likely plays an important role in flame propagation by transporting radical species along the longitudinal (laser propagation) axis of the flame, such that its separation can have a dramatic impact on subsequent flame propagation. This idea is also supported by Bradley *et al*.^26^ where, by comparing the propagation distances of the third lobe in combustible and pure air mixtures, it was concluded that the propagation of the third lobe is chemically enhanced. For the non-propagating case at ϕ = 0.6, one observes that once the third lobe becomes separated, the chemiluminescence signal decreases significantly even in the main kernel. This is likely indicative of the termination of chain branching chemical reactions and leads to complete extinction a few hundred microseconds later. The behaviors of third lobe separation and flame quenching are more readily apparent in the present chemiluminescence imaging (of OH*), since it provides direct chemical information, as compared to past work that has predominantly used shadowgraph or Schlieren, which probes variations in index-of-refraction (mainly due to gas heating)^24, 40–42^.

### Dual-Pulse Laser Ignition

**Figure 2:**
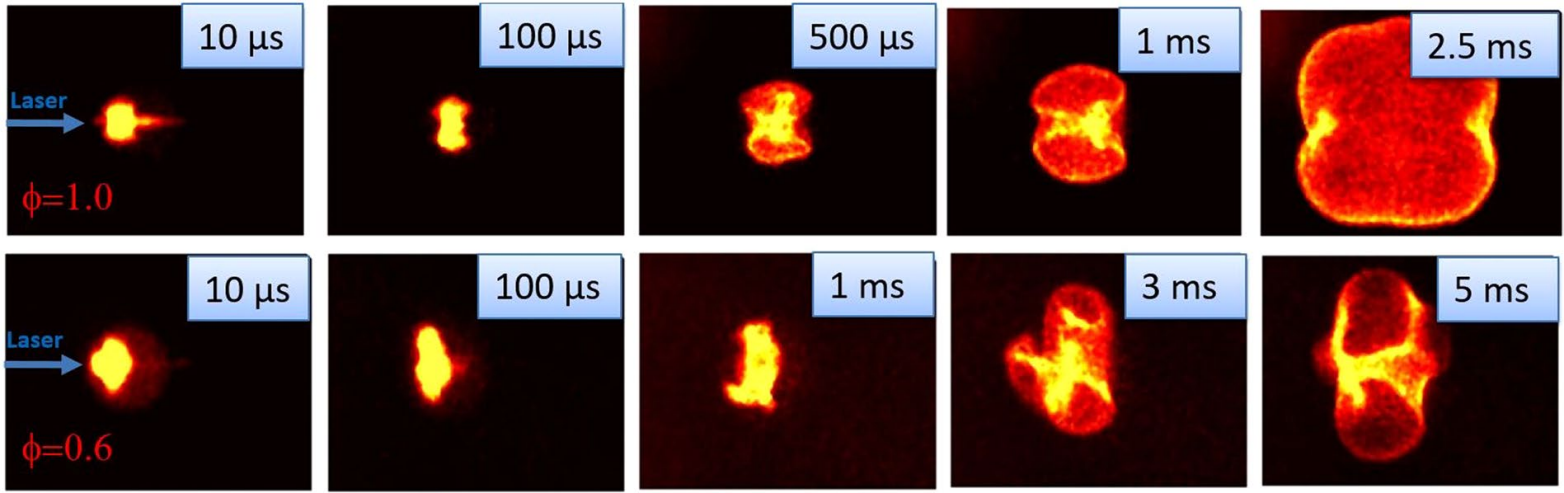
Flame kernel development during combustion of propane air mixtures at ϕ = 1.0 (top) and ϕ = 0.6 (bottom). Note that no third lobe is observed in this case. Each image frame has field of view: 22 mm × 16 mm.

Here we conduct a similar study using the dual-pulse technique with a combination of UV pre-ionization and NIR energy addition pulses. Results of OH* chemiluminescence imaging of the dual-pulse method are presented in Fig. 2. The laser energy for the UV pre-ionization pulse was 20 mJ while the NIR energy addition pulse was set to 40 mJ corresponding to a total delivered energy of 60 mJ (compared to 75 mJ for the single-pulse NIR laser spark ignition). The corresponding laser fluence are 260 J/cm^2^ for dual-pulse and 330 J/cm^2^ for the NIR laser spark ignition based on the focal spot diameter of 170 μm for both cases. For Fig. 2, the UV and NIR focal spots were axially overlapped along the beam path (zero offset) and the delay between the two pulses was set to 15 ns. Clearly, the flame dynamics are very different for the dual-pulse case as compared to single-pulse. We find that the dual-pulse technique allows one to suppress the formation of the third lobe by adjustment of the axial offset, which in turn has a significant impact on flame growth. For the stoichiometric case, i.e.ϕ = 1.0 in the top row of Fig. 2, the ignition conditions yield a quasi-spherical flame – quite different from its single-pulse counterpart (top row of Fig. 1). Moreover, the dual-pulse method, even with lower total energy, provides an extension of the lean limit as successful flame growth is observed for the ϕ = 0.6 (which extinguished with single-pulse). The suppression of the third lobe avoids the problems associated with its detachment as further discussed below.

### Combustion Efficiency

An investigation of the lean limit for propane-air mixtures ignited with both single- and dual-pulse techniques was performed by examining the efficiency of converting the fuel chemical energy into heat. The total available chemical energy of the fuel is computed in terms of the lower heating value (LHV) as:

1 $${E}_{fuel}={m}_{fuel}\times LHV$$

where *m*_*fuel*_ represents the mass of propane added into the chamber. The apparent heat release, *Q*, can be directly determined from the pressure time history and takes into account the heat losses to the wall (taken to be positive), ${\dot{Q}}_{wall}$, according to the following relation:

2 $$\frac{dQ}{dt}=\frac{1}{{\gamma }_{mix}-1}V\frac{dp}{dt}+{\dot{Q}}_{wall}$$

where the ratio of specific heats, *γ*_*mix*_, is computed at the average between the initial temperature and adiabatic flame temperature^13^. The rate of heat loss to the wall is estimated by analyzing the rate of pressure decay after the combustion event and is generally less than 20% of the total heat release (depending on the equivalence ratio). Finally, the combustion efficiency, *η*, is estimated as the ratio between the apparent heat release (obtained by temporal integration of Eq. 2) and the fuel energy content:

3 $$\eta =Q/{E}_{fuel}$$

**Figure 3:**
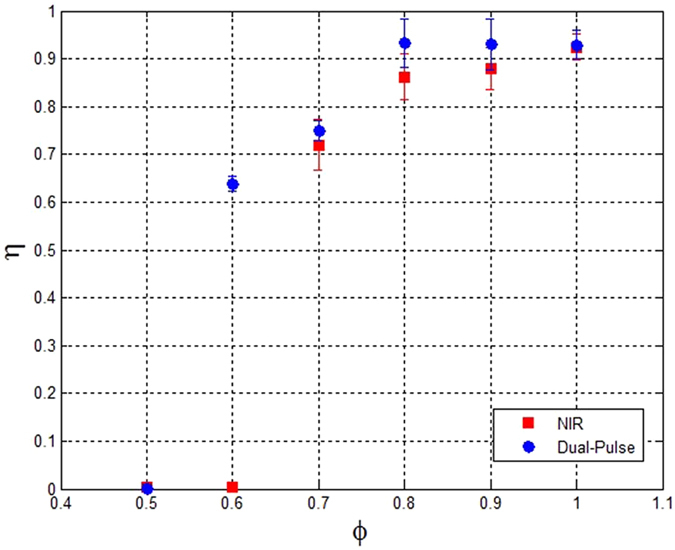
Combustion efficiency for NIR laser spark ignition (red) and Dual-Pulse (blue) at various propane-air equivalence ratios. Each point represents the average of multiple tests and the error bars are +/−one standard deviation.

Figure 3 shows the combustion efficiency for both methods as a function of equivalence ratio. The data points for near stoichiometric mixtures (ϕ = 0.8–1.0) represent the average of *η* from three repeated tests while for the leaner ratios (ϕ = 0.5–0.7) the test was repeated 10 times. For both methods, the efficiency drops at sufficiently lean conditions allowing identification of the lean limit. For increasingly lean conditions, the combustion efficiency of the single-pulse method is lower than for the dual-pulse case. These results demonstrate the ability of the dual-pulse method to extend the lean limit relative to conventional laser ignition.

### Effect of Spatial Offset on Dual-Pulse Ignition

**Figure 4:**
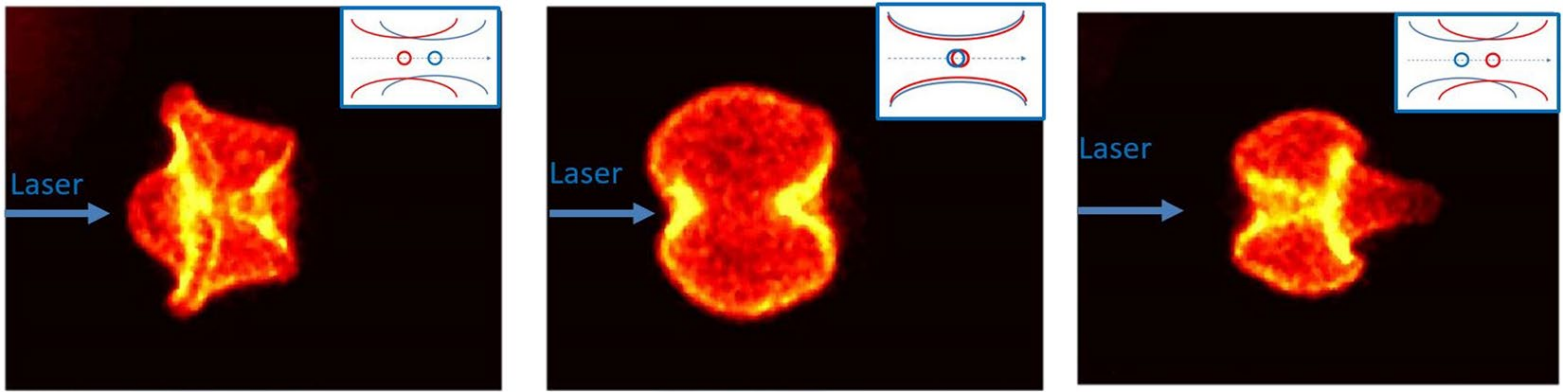
Dynamics of the flame kernel based on the spatial overlap between the two laser pulses. The images were all taken at delay of 1 ms with respect to the laser firing. Inserts at the top right corner shows the spatial overlap of the UV (blue) and NIR (red) beam waists. (The beams in the middle insert are laterally offset for visual clarity).

In the dual-pulse case, the formation of toroidal structures (and the associated vorticity) has been found to depend strongly on the spatial overlap and offset of the focal spots. Figure 4 shows how the flame dynamics are influenced by changing the axial offset between the focal spot positions of the two pulses (experimentally achieved with a translation stage for the focusing lens for the NIR beam). For the left image of Fig. 4 the NIR focal spot is 2 mm upstream of the UV, for the middle image the offset is zero (corresponding to the case presented in Figs 2 and 3), while for the right image the NIR focal spot is 2 mm downstream of the UV. For cases with an offset, a lobe structure tends to appear on the NIR side (i.e. towards the laser in the left image, and away from the laser in the right image). The flow field instabilities that accompany the recombination and plasma kernel cooling stages dictate the propagation direction of the flame. More specifically, depending if the NIR pulse is focused in front or behind the waist of the UV pre-ionization pulse, the counter-rotating vortices formed on either side of the plasma kernel are going to exhibit different strengths. From a fluid dynamics perspective, the differing strength is due to the fact that the epi-center of the (single) shock wave is always biased towards the location of the NIR beam waist (due to the non-uniform energy deposition which is strongest at the avalanche location). The differential vorticity leads to the generation of a third lobe propagating towards the left or right as shown in Fig. 4. If the beam waists of the NIR and UV pulses are overlapped along the laser propagation axis, the two counter-rotating vortices have equal strength and no third lobe is observed. We have also examined the absorbed pulse energies for the different offsets which we found to be constant (to within measurement error of ~5%). This is, of course, expected for the UV pulse, since its conditions are not changed by this offset, and the lack of change of the IR energy absorption is attributed to the fact that all offsets studied are still within the Rayleigh range of the UV beam (~3 cm). It is also noted that the total energy deposited in the dual-pulse case (~2 mJ absorbed due to UV plus ~13 mJ absorbed from NIR) is similar to that absorbed when the NIR pulse is applied on its own (~15 mJ absorbed due to the NIR only), suggesting that the lean limit is governed by the flame dynamics induced by the plasma rather than the amount of energy absorbed into the ignition kernel.

### Flame Speed Enhancement

**Figure 5:**
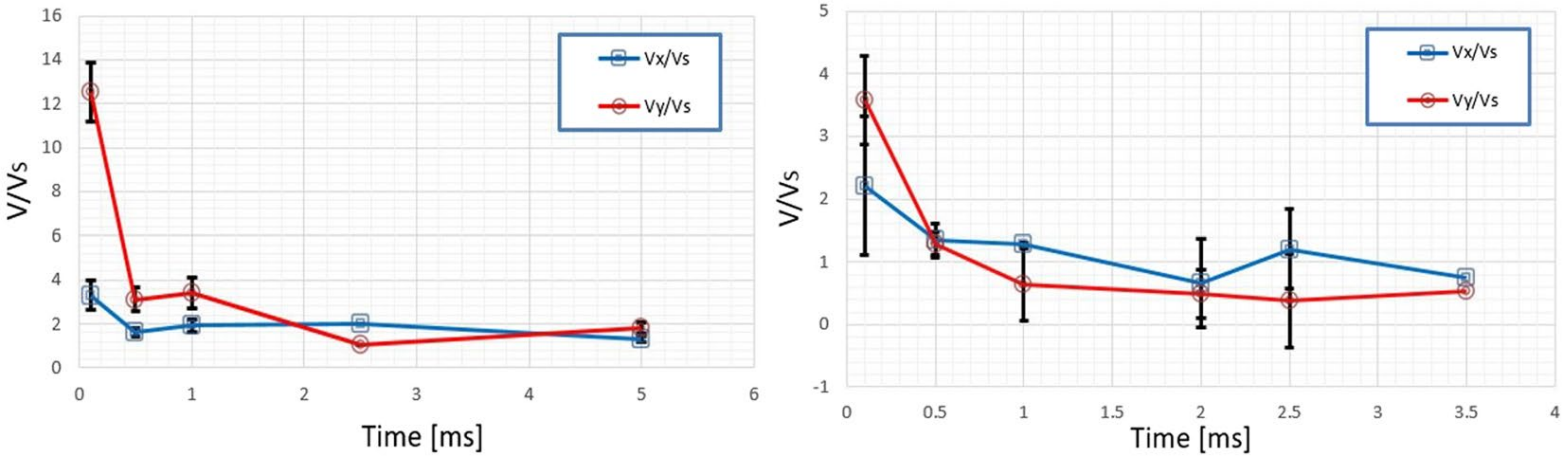
Normalized flame speed for a ϕ = 1.0 propane-mixture for the laser breakdown ignited mixtures (left) and dual-pulse (right). Error bars represent +/−one standard deviation.

To quantify the impact of the plasma induced vorticity on the flame propagation we examine the flame speeds in different directions, *V*_*x*_ and *V*_*y*_, where *x* is defined to be along the laser beam and *y* perpendicular to it. The analysis is based on temporal image sequences from the OH* chemiluminescence study. The flame speeds are determined from changes of the flame edge position relative to the center of the toroid. (The flame edge is indicated by the OH* chemiluminescence edge which is found from its maximum gradient.) The x- speed component considers the change in (lateral) flame edge position as it moves towards the laser, while the y-component is found from the furthest flame (transverse) edge away from the center line. The flame speed data presented in Fig. 5 is normalized by the theoretical unstretched laminar flame speed, *V*_*S*_, and plotted for both single- and dual-pulse ignition. For the stoichiometric case considered here, an unstretched laminar burning velocity of 0.3 m/s is used to compute the laminar flame speed^43^. For the single-pulse (NIR) case, shown in the left of Fig. 5, one observes that excessive stretching is present at early times. In particular, the flame speed in the *y*-direction is ~13 times higher than the unstretched laminar speed at early times. Conversely, as shown in the right of Fig. 5, the stretching effects exhibited by the dual-pulse are much lower (only~4 times larger than the laminar speed). These findings suggest that the third lobe separation (which severs the reaction zone) for NIR ignition at lean conditions results from excessive stretching. Therefore, it is reasonable to conclude that the dual-pulse technique extends the combustion lean limit by reducing flame stretch through suitable alignment of the two beams to suppress the third lobe.

### Summary

This study introduces a novel method for laser ignition using a dual-pulse pre-ionization technique. A comparative study between the well-established laser spark ignition and dual-pulse is presented. The results indicate that using the dual-pulse technique leads to a reduction of the lean limit for propane-air mixture and an increase in combustion efficiency. Additionally, the energy required for ignition for the dual-pulse method is lower (*E* = 60 mJ) compared to the NIR laser spark ignition (*E* = 75 mJ). A detailed study of the differing flame dynamics was also conducted using OH* chemiluminescence imaging. It is shown that the flame kernel develops as a toroidal structure that is governed by the fluid mechanics induced during the plasma recombination process. For lean mixture the third lobe (that points towards the laser beam) breaks from the main toroidal flame structure with negative effects on flame development. For the dual-pulse method, we show that the flame dynamics can be controlled by adjusting the axial offset of the UV and NIR beams resulting in reversal or elimination of the third lobe structure that typically points toward the laser. It is posited that the third lobe has important consequences towards flame growth and extinction.

## Methods

**Figure 6:**
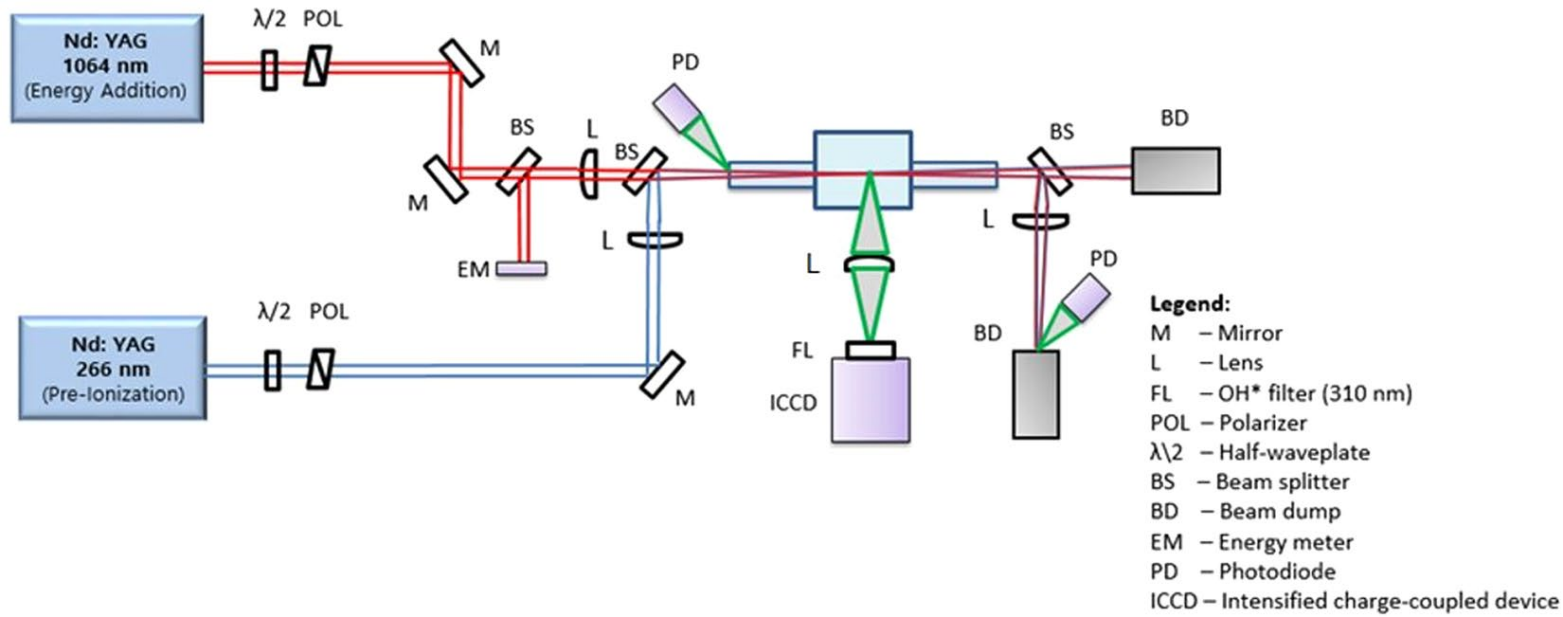
Optical layout used for the laser ignition experiments. Single-pulse laser ignition method uses solely the 1064 nm Nd:YAG, while the dual-pulse method uses overlapped beams from both the 266 nm and 1064 nm Nd:YAG lasers.

The experiments were conducted using the setup shown in Fig. 6. For dual-pulse experiments, the UV pre-ionization pulse was the fourth harmonic (λ = 266 nm) of an Nd:YAG (Continuum Powerlite 8010), while the energy addition pulse was the fundamental output (λ = 1064 nm) from a second Nd:YAG (New Wave Gemini PIV). Each beam has a variable attenuator, comprised of a half-wave plate and polarizer pair, to control laser energy. The 266 nm beam has 7 ns pulse duration and typical delivered energy of 20 mJ, while the 1064 nm beam has 10 ns pulse duration and typical delivered energy of 40 mJ (exact numbers are given with the experimental results). The two beams are spatially overlapped (with precision ~10 *μ*m) using a beam splitter and focused inside the combustion chamber using two *f* = 300 mm plano-convex lenses (one in each beam path). The focusing configuration yields beam diameters of 170 μm for both beams as measured using the 4-σ method. To vary the offset between the two beams in the axial direction (i.e. along the beam propagation direction), a translation stage was used to move the focusing lens of the 1064 nm beam. Both the beam waist axial overlap and waist diameters were determined experimentally using a beam profiler (Spiricon SP503). The combustion chamber has a central volume of ~0.195 liters and two side arms of length ~20 cm with 2.54 cm diameter circular windows for optical access. A series of photodiodes and energy meters are used to monitor laser pulse durations and pulse energies of each beam leg (i.e. UV and NIR) both before and after passage through the focal region and combustion chamber. For comparative experiment with conventional single-pulse laser ignition, the same 1064 nm laser is used without the accompanying 266 nm beam.

We record pressure data of combustion events using a pressure transducer (PCB: 113B24) mounted on the inner wall of the chamber. This information is used to determine the lean limit and the combustion efficiency in each test case. Additionally, chemiluminescence images of the OH* radical were acquired using an intensified charged coupled device (ICCD) camera (PCO dicam pro). The electronically excited hydroxyl radical is generated during the combustion of hydrocarbon fuels through the chain branching reaction: CH + O_2_ = OH* + CO. The excited OH* emits light at ~310 nm as it relaxes to the ground state^44^. For CL imaging of this transition a 310 nm bandpass filter (Andover: 310FS10-50, FWHM: 10 nm) was placed in front of the ICCD.

For the combustion experiments presented in this study, the combustible mixture consisted exclusively of propane-air at an initial pressure of 1 bar. Various equivalence ratios ranging from ϕ = 0.6–1.0 were tested with mixtures prepared inside the chamber based on partial pressures recorded from a gage (Omega DGP409) mounted downstream of the chamber valve. Once the chamber was filled, there was a 10 minute wait period to allow the fuel and air species to fully mix prior to ignition. Finally, after each experiment, the chamber was flushed with zero air and emptied to a pressure of <1 mbar (by connecting to a vacuum pump) to ensure any effects of residual combustion gases were eliminated.
